# Quantifying He fluxes from the mantle using multi-tracer data assimilation

**DOI:** 10.1098/rsta.2015.0288

**Published:** 2016-11-28

**Authors:** Reiner Schlitzer

**Affiliations:** Alfred Wegener Institute, Helmholtz-Center for Polar- and Marine Research, Am Alten Hafen 26, 27568 Bremerhaven, Germany

**Keywords:** GEOTRACES, helium isotopes, mid-ocean ridges, hydrothermal vents, micronutrients, data assimilation

## Abstract

A global, coarse-resolution ocean model previously fitted to geostrophic shear estimates and to data of 10 hydrographic parameters and tracers has been used to simulate the ^3^He and ^4^He distributions resulting from the release of mantle helium from mid-ocean ridges. The model is in very good agreement with ^14^C and chlorofluorocarbon data and has realistic global ocean overturning strength as well as water mass formation and transport rates. It is found that previously published global mantle ^3^He fluxes are too high by a factor of about 2. In the model, optimal agreement of modelled δ^3^He with data is achieved for a global flux of 450 ± 50 mol ^3^He yr^−1^. The formulation of He source strengths proportional to ridge spreading rates appears compatible with data. A model/data misfit analysis shows significant and large-scale δ^3^He underestimation in the southwestern Pacific centred over the Lau Backarc Basin (approx. 179° W/20° S). These misfits disappear in a set-up with 30 of the 450 mol yr^−1^ global total ^3^He flux released in the Lau Basin over a depth range between 1250 and 2500 m. Such He flux contributions are missing in present mantle He source compilations. Hydrothermal fluxes of other trace elements and isotopes (TEI) can be calculated from He fluxes on the basis of TEI : He ratios measured close to the sources.

This article is part of the themed issue ‘Biological and climatic impacts of ocean trace element chemistry’.

## 1. Introduction

**Figure 1.**
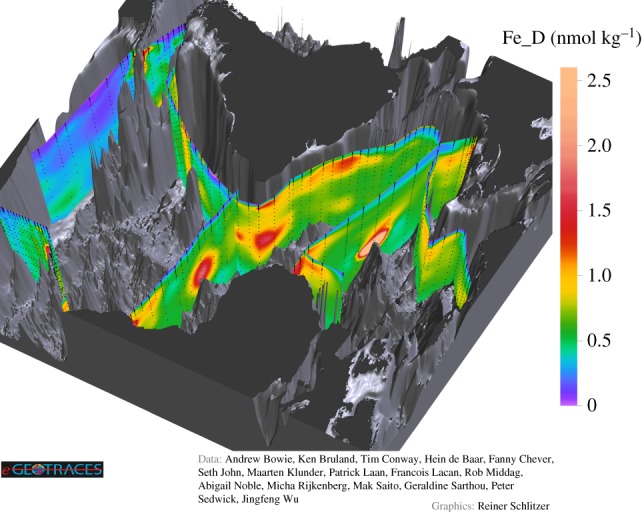
Concentration of dissolved Fe (in nanomoles per kilogram) along GEOTRACES sections in the Atlantic (from [1]). Note the plumes of high Fe concentrations where sections cross or come close to ocean ridges.

One of the exciting discoveries of the GEOTRACES observational programme are the pronounced deep-ocean trace element plumes originating from hydrothermal vents along geologically active ocean ridges found in all ocean basins. As an example, figure 1 [1] shows the distributions of dissolved Fe along six sections in the Atlantic. Clearly visible are plumes of very high Fe concentrations over the Mid Atlantic Ridge at 12° S [2] and 25° N [3] and over the Southwest Indian Ridge at 55° S [4]. The patch of high Fe waters in the tropical west Atlantic between 2000 and 3000 m [5] also seems to be caused by hydrothermal activity at the nearby ridge. High Fe plumes have also been discovered in the Arctic over the Gakkel Ridge [6], in the Indian Ocean over the Central Indian Ridge [7], and recently over the East Pacific Rise [8], where the plume extends westward over more than 4000 km. Other trace metals, such as Al, Mn and Zn, are also released at hydrothermal vents in large quantities and often show pronounced plumes over mid-ocean ridges.

It has been known since the 1960s that hydrothermal vents along spreading ocean ridges also release large amounts of primordial He originating from the Earth's mantle. Primordial He is enriched in the light isotope, ^3^He, and has ^3^He/^4^He isotopic ratios about a factor 8 higher than in atmospheric He [9,10]. Pronounced plumes of high He concentrations as well as δ^3^He isotopic signature have been observed in all ocean basins [8,11–21]. As a noble gas, He is a conservative tracer not undergoing chemical reactions. Therefore, shape and extent of the He plumes as well as the absolute concentrations in the plumes only depend on the source strengths at the hydrothermal vent sites and the direction and strength of the circulation transporting the He laterally and vertically away from the source. If the circulation is known from direct observations or modelling, mantle He source strengths can be determined from He data using He mass budget equations. Hydrothermal source strengths of other trace elements or isotopes (TEI) can then be calculated from TEI : He concentration ratios measured near the sources. Chemical reactions and transformation processes acting on TEIs after the release can, in principle, then be inferred and quantified using relative concentration differences between TEI and He. Therefore, the deep-ocean mantle He can be regarded as a reference tracer for other non-conservative TEIs also released by hydrothermal activity, and results obtained from the evaluation of He data help the interpretation of the more complicated biogeochemically active trace elements, such as Fe, Zn, Mn and Al.

Mantle He deep-ocean data have been used in many model studies to quantify source strengths at mid-ocean ridges and/or to validate model deep-ocean circulation fields. Craig *et al*. [22] used the limited number of ^3^He data available at the time in a simple box model of the global ocean to estimate a flux of mantle ^3^He from ocean ridges as 1070 ± 270 mol ^3^He yr^−1^. Later, [23] used a four-box model of the global ocean with water transports constrained by ^14^C data and obtained much smaller fluxes between 267 and 534 mol ^3^He yr^−1^. Dutay *et al*. [24] and Farley *et al*. [25] used global, medium resolution models that had widely been used for carbon cycle and other tracer studies to simulate mantle ^3^He distributions for the validation of the model's deep-ocean circulation. These models have mantle He sources along the mid-ocean ridges, with source strengths proportional to ridge spreading rates and a globally integrated ^3^He flux of 1000 mol yr^−1^ as in [22]. The simulated δ^3^He values were generally too high and agreement with data was only qualitative. Using various versions of the Princeton/GFDL model, [26] also found too high δ^3^He model values and proposed a reduced flux of mantle ^3^He from ocean ridges of 527 ± 102 mol ^3^He yr^−1^.

Here, the AWI Adjoint Tracer Model (AATM) previously fitted to geostrophic shear estimates and to the global data of 10 hydrographic parameters and tracers is applied to simulate the ^3^He and ^4^He distributions resulting from the release of mantle helium from mid-ocean ridges. The model is in very good agreement with ^14^C and chlorofluorocarbon (CFC) data and has realistic global ocean overturning strength as well as water mass formation and transport rates. Details of the deep-ocean model flows are in good agreement with the flow fields of [27] that are based on a careful analysis of geostrophic velocity estimates. The objectives are to quantify the mantle He sources along the mid-ocean ridges and study the effect of deep-ocean circulation in creating the observed δ^3^He plumes.

## 2. Data and model

Well-established methods for contamination-free sampling and reliable measurements of ^4^He concentration and the ^3^He/^4^He isotopic ratio in seawater have been in use for a long time. Therefore, now a large, publicly available high-quality He database exists that covers all oceans, provides dense coverage and reveals small- and large-scale features in the distributions. This is in contrast with many TEIs, which can only be sampled and measured properly since a few years, and for which only much smaller datasets are available [28]. This study uses public ^4^He concentration and δ^3^He isotope data downloaded from the CCHDO data centre (http://cchdo.ucsd.edu/; as of 2 November 2015). Data from [17] were added manually. Overall, this data collection contains 3199 stations with 42 525 ^4^He and 46 148 δ^3^He data values.

**Figure 2.**
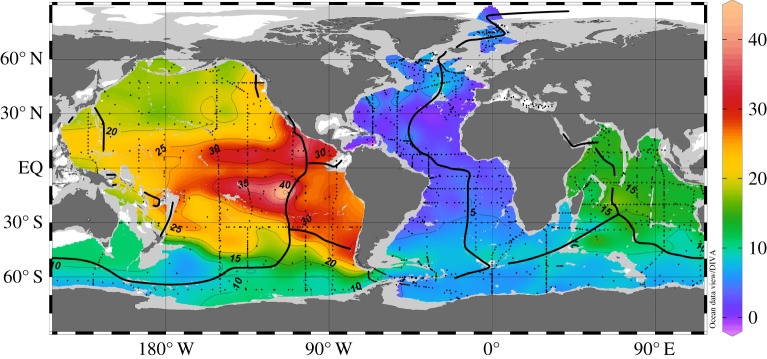
Distribution of observed δ^3^He (in per cent) in 2500 m depth. Black dots mark the positions of the measurements and black lines indicate the location of the mid-oceanic ridges, where mantle He is released into the seawater.

Figure 2 shows the distribution of δ^3^He at 2500 m depth. This layer is slightly above the average depth of mid-ocean-ridge crests and most clearly shows the effects of the mantle He sources. Mantle He is enriched by a factor of about 8 in ^3^He relative to atmospheric He [9,10], and seawater δ^3^He values are elevated near hydrothermal sources. There are large δ^3^He differences between ocean basins, with the Atlantic being lowest (approx. 5%), followed by intermediate values in the Indian Ocean (approx. 15%), and high values exceeding 20% in the Pacific. These differences are the consequence of differences in mantle He source strengths in the different basins, but also reflect water mass ages along the global conveyor belt path, with Atlantic deep water youngest and closest to atmospheric isotope values. Very high δ^3^He values above 30% in the low latitude East Pacific are created by known large mantle He sources along the East Pacific Rise, Chile Ridge, Galapagos Spreading Ridge and Juan de Fuca Ridge. Clearly visible are two separate westward spreading δ^3^He plumes south and north of the Equator. The cruise track of [8] coincides with the southern plume.

This study uses the AATM, a coarse-resolution global model with time-invariant circulation that has been fitted to hydrographic and tracer data by means of the adjoint method [29–31]. Radiocarbon and CFC data are included to guarantee realistic deep and bottom water transport rates and spreading pathways as well as a realistic strength of the global overturning circulation. It was shown [31] that AATM simultaneously produces global ocean distributions of hydrographic parameters and tracers that are in very good agreement with observations. In particular, the model correctly reproduces the deep-ocean radiocarbon field and ^14^C concentration gradients between different basins, suggesting that residence times in the deep ocean and deep-ocean water mass transport are realistic.

**Figure 3.**
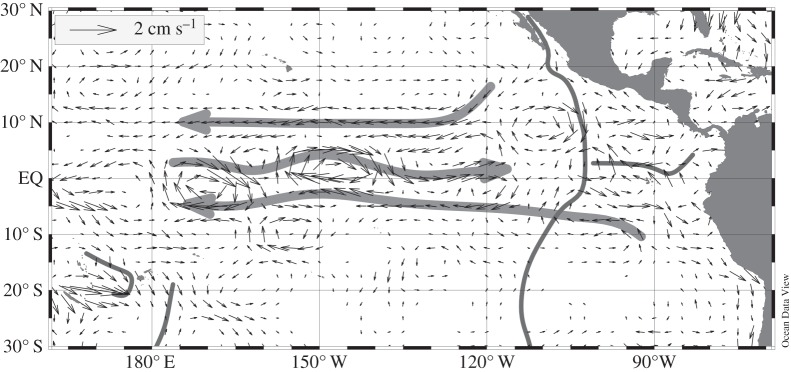
Model flow velocities in the depth range between 2000 and 3500 m in the tropical eastern Pacific. The thick grey arrows highlight the sequence of westward flows south and north of the Equator and eastward flow on the Equator. The thin grey lines indicate the position of mid-ocean ridges.

Figure 3 shows the average model flow field in the low latitude eastern Pacific in the depth range between 2000 and 3500 m depth, e.g. the layer receiving most of the mantle He in this region. The circulation field is dominated by a succession of zonally oriented flows of about 1 cm s^−1^, with separate westward branches south and north of the Equator and eastward flows on the Equator. This pattern is in good agreement with the dynamic height analysis of [27], which also shows predominantly zonal flows with the same orientations.

The set-up of the AATM mantle He simulations follows the OCMIP protocol of Dutay *et al.* [24]. He is injected along mid-ocean ridges at rates proportional to the local ridge spreading rates and a globally integrated flux of 1000 mol ^3^He yr^−1^. The centre of the release is 300 m above ridge crests to account for buoyancy effects of the released hot fluids. The ^3^He/^4^He isotopic ratio of the hydrothermal He sources is a factor 8 higher than for atmospheric He. Concentration boundary conditions are applied at the ocean surface based on evidence from observations: ^4^He is slightly supersaturated (102.5%) relative to solubility equilibrium at local temperatures [32], and a spatially uniform δ^3^He value of −1% is applied. Using this set-up, AATM was run to simulate separate fields of ^3^He and ^4^He, which were then combined to obtain model δ^3^He values to be compared with observations.

It is important to note that the present model runs do not include tritium decay as additional source of ^3^He. In the real ocean tritiugenic ^3^He concentrations are significant in the upper water column, while in the deep ocean, the focus of this study, this contribution is generally very small. Here, the quantitative model/data misfit analysis (see below) is confined to regions and depths where tritium as well as tritiugenic ^3^He concentrations are negligible.

## 3. Results and discussion

**Figure 4.**
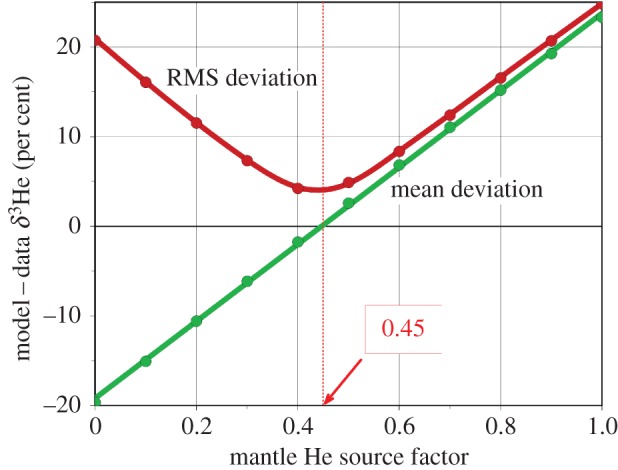
Mean and root-mean-square (RMS) model minus data differences in the Pacific between 1500 and 4500 m depth as a function of a global mantle He source factor. A source factor 1 represents a globally integrated source of 1000 mol ^3^He yr^−1^, and a factor 0 represents zero mantle He flux. Optimal agreement between model and observations is obtained for a global mantle ^3^He source of 1000 mol yr^−1^.

The initial model simulation with a total mantle source of 1000 mol ^3^He yr^−1^ yielded δ^3^He values systematically larger than observed and the run was rejected because of incompatibility with data. A sequence of sensitivity runs with reduced mantle He sources were conducted by applying a spatially constant reduction factor between 1 (flux of 1000 mol ^3^He yr^−1^) and 0 (no mantle He release). The resulting mean and root-mean-square (RMS) model minus data differences in the Pacific Ocean between 1500 and 4500 m depth are shown in figure 4 as a function of the source reduction factor. Optimal agreement between model and data is achieved for a global mantle He source of 450 mol ^3^He yr^−1^. At this rate, the mean difference is zero and the RMS difference (4%) is minimal and amounts to about two times the measurement error only. Taking into account the slope of the mean difference curve in figure 4 and applying an acceptable mean difference limit of ±2.5% leads to an error estimate for the mantle ^3^He source of 50 mol ^3^He yr^−1^.

The AATM results are consistent with the Dutay *et al.* [24] and Bianchi *et al.* [26] simulations, who also found too high simulated δ^3^He values with the 1000 mol ^3^He yr^−1^ source strength. The AATM optimal source strength of 450 ± 50 mol ^3^He yr^−1^ agrees within error bars with [23,26]. The alternative explanation that the too high model δ^3^He values for the original source strength are caused by too sluggish deep water circulation can be rejected because of the excellent agreement of the AATM model with CFC and ^14^C data. Reducing the deep Pacific residence time in the model by invigorating deep flows on the basin scale in the model would lead to unrealistically high deep-ocean CFC and ^14^C concentrations.

**Figure 5.**
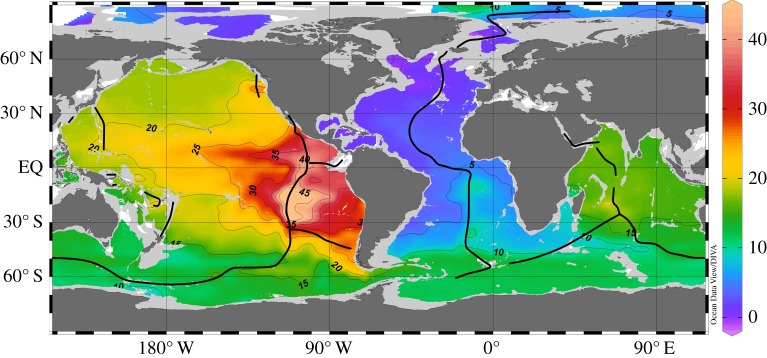
Model simulated distribution of δ^3^He (in per cent) in 2500 m depth for a globally integrated mantle ^3^He source of 450 mol yr^−1^ that yields optimal model/data agreement. Black dots mark the model grid and black lines indicate the location of the mid-oceanic ridges, where mantle He is released into the seawater.

The model δ^3^He distribution at 2500 m depth for the optimal source strength is shown in figure 5. This distribution should be compared with the corresponding observations in figure 3. The model correctly reproduces the different average δ^3^He levels in the Atlantic, Indian and Pacific Oceans. As in the observations, the model shows highest δ^3^He values over the East Pacific Rise at two locations south and north of the Equator. From there, two separate δ^3^He plumes extend westward into the central and western Pacific south and north of the Equator. δ^3^He values at the Equator are much lower compared with plume values. All these features are in good agreement with observations. Previous He model simulations failed to reproduce the two separate westward spreading δ^3^He plumes [24,26] and underestimated the spatial scale of the westward transport away from the East Pacific Rise into the western Pacific, most probably because of deficiencies in the deep-ocean circulation fields. In the AATM, the separate plumes are a direct consequence of the specific flow pattern (figure 3) consisting of strong westward flows south and north of the Equator and eastward flows at the Equator. While reproducing the data better than previous models, AATM still underestimates the scales of the westward transport and overestimates δ^3^He values at the source locations over the East Pacific Rise, especially south of the Equator. A flow pattern as in figure 3 but with more intense westward and eastward velocities would probably lead to an even better reproduction of the observed δ^3^He field, and at the same time leave the simulated ^14^C distribution largely unchanged, because of the quite homogeneous radiocarbon distribution at these depths.

**Figure 6.**
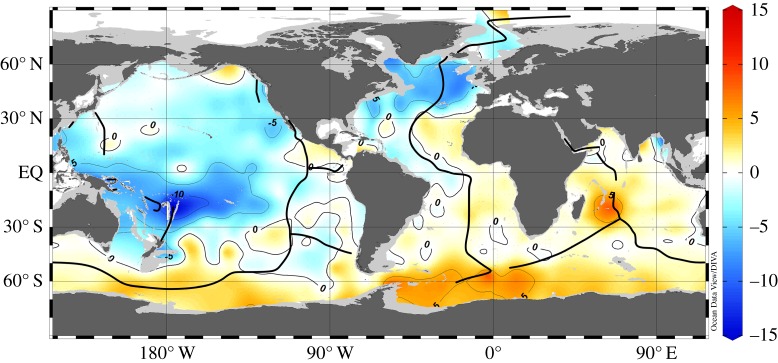
Vertical profiles of the model minus data δ^3^He differences (in per cent) in the Lau Backarc Basin area. Clearly visible is the model δ^3^He underestimation of up to −15% in the depth range between 1000 and 3000 m depth.

**Figure 7.**
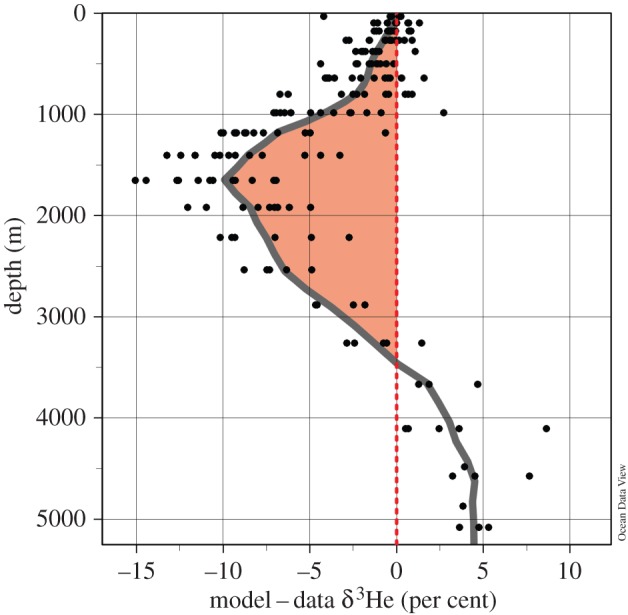
Model minus data δ^3^He differences (in per cent) in 1650 m depth for the solution with a globally integrated mantle ^3^He source of 450 mol yr^−1^. Clearly visible is a large-scale and significant model underestimation centred near 178° W/20° S in the Lau Backarc Basin.

Another quite large model/data misfit can be located in the area of the Lau Backarc Basin (179° W/20° S) in the southwestern Pacific, where the model underestimates δ^3^He values at 2500 m depth by about 8%. Profiles of the model/data misfit for model grid points in a 20° × 20° wide box centred over the Lau Basin (figure 6) reveal that the model δ^3^He underestimation extends over a wide depth range with largest deficiencies at 1650 m depth, where the model δ^3^He underestimation reaches up to 15%. A global map of model/data misfits at the 1650 m depth layer is shown in figure 7. The largest feature is the model δ^3^He underestimation in the Lau Backarc Basin with an average deficit of 10% within the basin area and deficits of more than 5% in a very large region covering the entire southwestern Pacific between 40° S and the Equator. The very large extent of the deficit region, in terms of both geographical area as well as depth space, clearly indicates an additional mantle He source in the Lau Basin not yet accounted for in the latest He source compilation. Only in the last 10 years has the Lau Basin been identified as a hydrothermally very active backarc spreading centre [16,33], and it is not surprising that its role as significant mantle He source was not properly taken into account previously.

Other, quite large model/data misfits in figure 7 are the much localized δ^3^He overestimation over the Central Indian Ridge near 60° E/20° S and the large-scale δ^3^He underestimation in the north and northwest Atlantic. The Indian Ocean feature is probably caused by a slightly too large assumed mantle He source strength at the particular location. The larger-scale model/data difference in the North Atlantic appears because considerable fractions of the observed ^3^He concentrations in this region and depth range are not of hydrothermal origin, but instead produced by the decay of tritium present in upper North Atlantic Deep Water found at this depth [34]. The AATM He simulations do not take into account ^3^He production by tritium decay and therefore do not include this component.

A series of sensitivity runs has been conducted to determine the required mantle He flux in the Lau Backarc Basin that would eliminate the systematic δ^3^He underestimation in the southwestern Pacific observed in figures 6 and 7. The best agreement with data was found with a Lau Backarc Basin source of 30 ± 5 mol ^3^He yr^−1^ (released in the depth range between 1250 and 2500 m) and a reduced source from the other mid-ocean ridges of 420 mol ^3^He yr^−1^, leaving the global ocean mantle He source unchanged at 450 mol ^3^He yr^−1^ to maintain the zero mean misfit. Adding the Lau Basin He sources improves agreement with data considerably. With this particular set-up, the mean model minus data misfit in the Lau Basin region (depth interval between 1000 and 3000 m) is reduced from −8.0 to −1.9% and the Pacific-wide RMS difference drops from 4.0 to 3.4%. The mean difference for the entire Pacific between 1500 and 4500 m remains close to zero. The estimated ^3^He source for the Lau Backarc Basin represents almost 7% of the total global flux, making the Lau Basin a major source region for the global marine mantle ^3^He budget.

**Figure 8.**
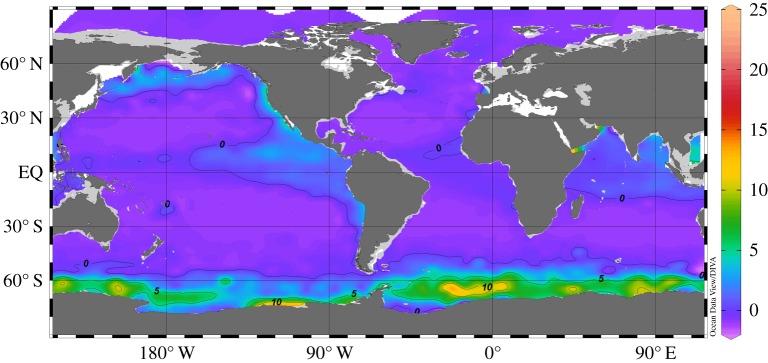
Map of simulated δ^3^He (in per cent) in 150 m depth, just below the euphotic zone. Most of the mantle He released in the deep ocean reaches the surface ocean in a continuous belt around the Antarctic continent.

Figure 8 shows the simulated δ^3^He just below the euphotic zone at 150 m depth and reveals the regions in the world ocean where mantle He is reaching the productive surface layer of the ocean. Most of the mantle He is found in the Southern Ocean around the Antarctic continent. Smaller fractions of the mantle He are seen in the north Pacific, eastern and tropical Pacific and in the tropical Indian Ocean. All these regions are well-known upwelling areas, and (because of the upwelling) all these regions show significant levels of macronutrients in the surface water, such as silicate, nitrate or phosphate. Other conservative substances released at hydrothermal vents together with the He are expected to reach the ocean surface in the same areas.

The data in [8] show that dissolved Fe released at hydrothermal vents along the East Pacific Rise is transported over large, basin-scale distances, suggesting a quasi-conservative behaviour of this important micronutrient in the water column. Presently, the underlying processes are not fully known or understood; however, it seems possible that significant fractions of the hydrothermal Fe actually do reach the ocean surface. According to figure 8, this would happen predominantly in the Southern Ocean, where the upwelled hydrothermal Fe could play an important role in stimulating surface ocean productivity [35].

## 4. Conclusion

The AATM has been applied successfully to simulate the ^3^He and ^4^He distributions in the global ocean resulting from the flux of mantle He released at hydrothermal vents along mid-ocean ridges. AATM is able to closely reproduce δ^3^He observations in all ocean basins. Mean and RMS model minus data differences of the optimal model simulation in the area most affected by mantle He release (e.g. Pacific between 1500 and 4500 m depth) amount to 0.6 ± 3.4%, with the RMS difference only about a factor 2 larger than the δ^3^He measurement error. Interbasin δ^3^He differences between Atlantic, Indian and Pacific are reproduced correctly, suggesting that the split of flux contributions from the different ocean basins and the assumption of proportionality between He flux and ridge spreading rate appear valid.

In the Pacific, the AATM reproduces the westward spreading of the He released along the East Pacific Rise much better compared with previous studies, although westward transports still appear to be too weak, especially south of the Equator. For the first time, AATM is able to reproduce smaller-scale details of the westward spreading, such as the two separate plumes south and north of the Equator. This is seen as a consequence of the predominantly zonal model deep-ocean flow pattern consisting of quite strong westward flows south and north of the Equator and an eastward counter-current along the Equator. This flow pattern is in very good agreement with the data-based analysis of the dynamic height in the deep Pacific in [27]. The inclusion of geostrophic shear constraints in the AATM optimization procedure is seen as very important measure for establishing realistic deep-ocean flows.

The AATM He simulations clearly show that the previously used global mantle ^3^He flux of 1000 mol yr^−1^ [22,24,25] is incompatible with He data and too high. AATM sensitivity runs show that a reduced mantle ^3^He flux of 450 ± 50 mol yr^−1^ is sufficient to achieve a mean model/data difference of zero. The AATM model is in excellent agreement with ^14^C data [31] and has realistic deep-ocean residence times. Therefore, the alternative hypothesis that the δ^3^He overestimation with the original, high ^3^He flux is a result of too sluggish deep circulation and too large residence times is rejected. Invigorating the deep circulation on basin scales would lead to violations of the ^14^C data constraints.

A misfit analysis of the AATM simulations using the He flux set-up in [24] and [26] shows significant and large-scale δ^3^He underestimation in the southwestern Pacific centred in the Lau Backarc Basin (179° W/20° S) area. These misfits are observed over a wide depth range between 1250 and 3000 m, suggesting a quite large mantle He source in this area not yet accounted for. In the model, the misfit can be eliminated by redistributing 30 of the 450 mol yr^−1^ global total ^3^He flux into the Lau Basin, released over a depth range between 1250 and 2500 m. The total global flux of 450 mol ^3^He yr^−1^ has to be maintained to ensure zero mean model/data differences. Many of the hydrothermal vents in the Lau backarc system have only been discovered in the last decade [16,33], and it is not surprising that its role as significant mantle He source was not taken into account previously. The results of this study call for an update of the present mantle He source compilations.

The model results for the mantle He fluxes from hydrothermal vents can be applied for the estimation of other TEI fluxes, such as Fe, Mn and Al, using TEI : He concentration ratios measured near the hydrothermal sources [36]. In addition, comparison of shape and spatial extent of observed TEI plumes with the documented δ^3^He plumes can provide quick indications of the importance of non-conservative processes for a given TEI.

## Data accessibility

The He data used in this study are accessible at http://cchdo.ucsd.edu/. He model simulations and codes can be obtained from the author.

## References

[1] Schlitzer R 2015 eGEOTRACES—electronic atlas of GEOTRACES sections and animated 3D scenes. See http://www.egeotraces.org.

[2] Saito MA, Noble AE, Tagliabue A, Goepfert TJ, Lamborg CH, Jenkins WJ 2013 Slow-spreading submarine ridges in the South Atlantic as a significant oceanic iron source. Nat. Geosci. 6, 775–779. (10.1038/ngeo1893)

[3] Hatta M, Measures CI, Wu J, Roshan S, Fitzsimmons JN, Sedwick P, Morton P 2015 An overview of dissolved Fe and Mn distributions during the 2010–2011 U.S. GEOTRACES north Atlantic cruises: GEOTRACES GA03. Deep-Sea Res. II Top. Stud. Oceanogr. 116, 117–129. (10.1016/j.dsr2.2014.07.005)

[4] Klunder MB, Laan P, Middag R, De Baar HJW, van Ooijen JC 2011 Dissolved iron in the Southern Ocean (Atlantic sector). Deep-Sea Res. II Top. Stud. Oceanogr. 58, 2678–2694. (10.1016/j.dsr2.2010.10.042)

[5] Rijkenberg MJA, Middag R, Laan P, Gerringa LJA, van Aken HM, Schoemann V, de Jong JTM, de Baar HJW 2014 The distribution of dissolved iron in the West Atlantic Ocean. PLoS ONE 9, e101323 (10.1371/journal.pone.0101323) 24978190 PMC4076309

[6] Klunder MB, Laan P, Middag R, de Baar HJW, Bakker K 2012 Dissolved iron in the Arctic Ocean: important role of hydrothermal sources, shelf input and scavenging removal. J. Geophys. Res. Oceans 117, C04014 (10.1029/2011JC007135)

[7] Nishioka J, Obata H, Tsumune D 2013 Evidence of an extensive spread of hydrothermal dissolved iron in the Indian Ocean. Earth Planet. Sci. Lett. 361, 26–33. (10.1016/j.epsl.2012.11.040)

[8] Resing JA, Sedwick PN, German CR, Jenkins WJ, Moffett JW, Sohst BM, Tagliabue A 2015 Basin-scale transport of hydrothermal dissolved metals across the South Pacific Ocean. Nature 523, 200–203. (10.1038/nature14577) 26156374

[9] Kurz MD, Jenkins WJ, Schilling JG, Hart SR 1982 Helium isotopic variations in the mantle beneath the central North Atlantic Ocean. Earth Planet. Sci. Lett. 58, 1–14. (10.1016/0012-821X(82)90099-1)

[10] Lupton JE, Craig H 1975 Excess ^3^He in oceanic basalts: evidence for terrestrial primordial helium. Earth Planet. Sci. Lett. 26, 133–139. (10.1016/0012-821X(75)90080-1)

[11] Clarke WB, Beg MA, Craig H 1969 Excess ^3^He in the sea: evidence for terrestrial primodal helium. Earth Planet. Sci. Lett. 6, 213–220. (10.1016/0012-821X(69)90093-4)

[12] Jamous D, Mémery L, Andrié C, Jean-Baptiste P, Merlivat L 1992 The distribution of helium 3 in the deep western and southern Indian Ocean. J. Geophys. Res. 97, 2243–2250. (10.1029/91JC02062)

[13] Jean-Baptiste P, Mantisi F, Pauwels H, Grimaud D, Patriat P 1992 Hydrothermal ^3^He and manganese plumes at 19° 29'S on the Central Indian Ridge. Geophys. Res. Lett. 19, 1787–1790. (10.1029/92GL00577)

[14] Lupton J 1998 Hydrothermal helium plumes in the Pacific Ocean. J. Geophys. Res. 103, 15 853–15 868. (10.1029/98JC00146)

[15] Lupton JE 1976 The ^3^He distribution in deep water over the Mid-Atlantic Ridge. Earth Planet. Sci. Lett. 32, 371–374. (10.1016/0012-821X(76)90077-7)

[16] Lupton JE, Arculus RJ, Resing J, Massoth GJ, Greene RR, Evans LJ, Buck N 2012 Hydrothermal activity in the Northwest Lau Backarc Basin: evidence from water column measurements. Geochem. Geophys. Geosyst. 13, Q0AF04 (10.1029/2011GC003891)

[17] Lupton JE, Craig H 1981 A major helium-3 source at 15°S on the East Pacific Rise. Science 214, 13–18. (10.1126/science.214.4516.13) 17802550

[18] Lupton JE, Graham DW, Delaney JR, Johnson HP 1993 Helium isotope variations in Juan De Fuca Ridge basalts. Geophys. Res. Lett. 20, 1851–1854. (10.1029/93GL01271)

[19] Lupton JE, Weiss RF, Craig H 1977 Mantle helium in hydrothermal plumes in the Galapagos Rift. Nature 267, 603–604. (10.1038/267603a)

[20] Takahata N, Agarwal M, Nishizawa M, Shirai K, Inoue Y, Sano Y 2005 Helium-3 plume over the East Pacific Rise at 25°S. Geophys. Res. Lett. 32, L11608 (10.1029/2005GL023076)

[21] Winckler G, Newton R, Schlosser P, Crone TJ 2010 Mantle helium reveals Southern Ocean hydrothermal venting. Geophys. Res. Lett. 37, L05601 (10.1029/2009gl042093)

[22] Craig H, Clarke WB, Beg MA 1975 Excess ^3^He in deep water on the East Pacific Rise. Earth Planet. Sci. Lett. 26, 125–132. (10.1016/0012-821X(75)90079-5)

[23] Jean-Baptiste P 1992 Helium-3 distribution in the deep world ocean. In Isotopes of noble gases as tracers in environmental studies (ed. Rozanski K), pp. 219–240. Vienna, Austria: International Atomic Energy Agency.

[24] Dutay JC et al. 2004 Evaluation of OCMIP-2 ocean models’ deep circulation with mantle helium-3. J. Mar. Syst. 48, 15–36. (10.1016/j.jmarsys.2003.05.010)

[25] Farley KA, Maier-Reimer E, Schlosser P, Broecker WS 1995 Constraints on mantle ^3^He fluxes and deep-sea circulation from an oceanic general circulation model. J. Geophys. Res. 100, 3829–3839. (10.1029/94JB02913)

[26] Bianchi D, Sarmiento JL, Gnanadesikan A, Key RM, Schlosser P, Newton R 2010 Low helium flux from the mantle inferred from simulations of oceanic helium isotope data. Earth Planet. Sci. Lett. 297, 379–386. (10.1016/j.epsl.2010.06.037)

[27] Reid JL 1997 On the total geostrophic circulation of the Pacific Ocean: flow patterns, tracers, and transports. Prog. Oceanogr. 39, 263–352. (10.1016/S0079-6611(97)00012-8)

[28] Mawji E et al. 2015 The GEOTRACES Intermediate Data Product 2014. Mar. Chem. 177, 1–8. (10.1016/j.marchem.2015.04.005)

[29] Schlitzer R 2002 Carbon export fluxes in the Southern Ocean: results from inverse modeling and comparison with satellite based estimates. Deep-Sea Res. II 49, 1623–1644. (10.1016/S0967-0645(02)00004-8)

[30] Schlitzer R 2004 Export production in the Equatorial and North Pacific derived from dissolved oxygen, nutrient and carbon data. J. Oceanogr. 60, 53–62. (10.1023/B:JOCE.0000038318.38916.e6)

[31] Schlitzer R 2007 Assimilation of radiocarbon and chlorofluorocarbon data to constrain deep and bottom water transports in the world ocean. J. Phys. Oceanogr. 37, 259–276. (10.1175/JPO3011.1)

[32] Weiss RF 1971 Solubility of helium and neon in water and seawater. J. Chem. Eng. Data 16, 235–241. (10.1021/je60049a019)

[33] Beaulieu SE, Baker ET, German CR 2015 Where are the undiscovered hydrothermal vents on oceanic spreading ridges? Deep-Sea Res. II Top. Stud. Oceanogr. 121, 202–212. (10.1016/j.dsr2.2015.05.001)

[34] Jenkins WJ, Rhines PB 1980 Tritium in the deep north Atlantic Ocean. Nature 286, 877–880. (10.1038/286877a0)

[35] Tagliabue A et al. 2010 Hydrothermal contribution to the oceanic dissolved iron inventory. Nat. Geosci. 3, 252–256. (10.1038/ngeo818)

[36] German CR et al. 2016 Hydrothermal impacts on trace element and isotope ocean biogeochemistry. Phil. Trans. R. Soc. A 374, 20160035 (10.1098/rsta.2016.0035) 29035265 PMC5069535

